# Lung ultrasound for neonatal respiratory distress in a resource-limited county hospital: a pilot study

**DOI:** 10.1186/s12887-026-06784-9

**Published:** 2026-03-26

**Authors:** Xingyu Zhu, Yunrui Zhuang, Kaili Qian, Fengfang Li

**Affiliations:** 1 ¹Department of Neonatology, Tongxiang Maternity and Child Health Care Hospital, Jiaxing, 314505 China; 2 ²Department of Ultrasound, Tongxiang Maternity and Child Health Care Hospital, Jiaxing, China

**Keywords:** Respiratory distress syndrome, Neonates, Lung ultrasound, Triage, Resource-limited setting

## Abstract

**Background:**

While tertiary centers have widely adopted lung ultrasound (LUS) as a diagnostic alternative to chest X-ray (CXR), recent evidence emphasizes its growing prognostic utility. This pilot study investigated the feasibility and clinical utility of LUS aeration scores in triage decisions within a resource-limited county-level hospital.

**Methods:**

This prospective pilot observational study enrolled neonates exhibiting signs of respiratory distress within 24 h of birth, employing a symptom-driven approach without predefined gestational age restrictions. Bedside LUS and CXR were performed within the first two hours of non-invasive respiratory support, prior to surfactant administration. LUS aeration scores were calculated using a standardized six-zone protocol (score range: 0–18). Following respiratory distress syndrome (RDS) diagnosis based on clinical and sonographic criteria, we evaluated the predictive performance of a predefined high-risk threshold (LUS aeration score ≥ 10) for triage outcomes (transferred vs. locally managed).

**Results:**

A total of 34 neonates (gestational age range: 30 to 40 + 2 weeks) were enrolled, of whom 17 (50%) were diagnosed with RDS based on composite clinical and sonographic criteria. Within the RDS subgroup, 6 neonates (35%) required tertiary transfer due to severe clinical progression (e.g., requirement for invasive mechanical ventilation), while 11 (65%) were successfully managed locally. Transferred neonates showed a non-significant trend toward higher baseline LUS aeration scores than locally managed peers (median 12 vs. 10, *P* = 0.242). However, when applying a predefined threshold (LUS aeration score ≥ 10), the score yielded a sensitivity of 83.3% and a negative predictive value (NPV) of 80.0% for predicting tertiary transfer.

**Conclusions:**

Bedside LUS is a feasible tool for assessing the severity of neonatal respiratory distress in resource-limited settings. Specifically, the promising negative predictive value of the LUS aeration score provides an objective basis and clinical confidence for managing low-risk neonates locally, thereby optimizing regional healthcare resources. Further large-scale studies are needed to validate these preliminary findings.

**Supplementary Information:**

The online version contains supplementary material available at 10.1186/s12887-026-06784-9.

## Introduction

Neonatal respiratory distress (RD) is one of the most common and critical clinical presentations evaluated in primary care settings. Among its etiologies, respiratory distress syndrome (RDS) remains a leading cause of morbidity in preterm infants [1], requiring prompt and specific interventions such as exogenous surfactant replacement. While tertiary centers have widely adopted lung ultrasound (LUS) as a radiation-free diagnostic alternative to chest X-ray (CXR), recent high-level evidence emphasizes its prognostic and predictive utility. Systematic reviews have validated LUS aeration scores for predicting early surfactant requirements [2, 3] and forecasting bronchopulmonary dysplasia (BPD) in extremely preterm infants [4, 5]. However, the translation of these predictive applications to non-tertiary centers remains limited. In China, the management of neonates is increasingly shifting towards county-level hospitals [6], which often face logistical barriers such as a lack of 24-hour radiology services and the risks associated with transporting unstable neonates [7].

Although LUS diagnostic features for RDS are well-validated, evidence regarding the triage utility of LUS aeration scores in primary care is scarce. Therefore, this pilot study aimed to evaluate the feasibility of implementing bedside LUS in a county-level hospital and to investigate the role of the LUS aeration score in facilitating triage decision-making.

## Materials and methods

### Study design and population

This single-center prospective observational study was conducted in the Neonatal Intensive Care Unit (NICU) of a county-level hospital in Zhejiang Province, China, following local ethics approval and parental informed consent. We employed a consecutive sampling approach, concluding enrollment once a sufficient exploratory sample size was achieved. There were no gestational age restrictions. Antenatal corticosteroid (ACS) exposure was defined as any maternal dose prior to delivery, and small for gestational age (SGA) as birth weight below the 10th percentile [8].

Signs of respiratory distress were objectively defined by at least two of the following within 24 h of birth: (1) tachypnea (> 60 breaths/min); (2) increased work of breathing (e.g., retractions, grunting, flaring); and (3) increased oxygen requirement (room-air cyanosis or SpO_2_ < 90% without supplemental support) [9]. Experienced neonatal pediatricians performed all initial clinical assessments. Exclusion criteria included known or suspected congenital cardiopulmonary malformations, the need for immediate cardiopulmonary resuscitation or endotracheal intubation, and surfactant administration in the delivery room.

## LUS protocol

Bedside LUS was performed by three attending ultrasound physicians using a Philips EPIQ 5 system with a high-frequency linear probe (5–12 MHz). Operators were strictly blinded to clinical data and baseline CXR findings. All scans were completed within the first two hours of non-invasive support and strictly before exogenous surfactant administration. A standardized 6-zone scanning protocol was employed as previously described [10, 11]. Each zone was scored 0–3 (0: normal A-lines; 1: ≥3 well-spaced B-lines; 2: coalescent B-lines with or without subpleural consolidation; 3: extended consolidation), yielding a maximum total score of 18 (Supplementary Figure). Aligning with the 2025 ESICM-ESPNIC consensus [12], this metric is termed the “LUS aeration score”.

## Diagnostic criteria

RDS was diagnosed based on characteristic clinical signs and compatible lung ultrasound (LUS) features, including consolidation with air bronchograms, pleural line abnormalities, and the absence of A-lines. Typical radiographic findings (ground-glass opacity and air bronchograms) were considered supportive [9].

## Clinical management and transfer criteria

Initial respiratory support was exclusively provided via nasal continuous positive airway pressure (nCPAP) or high-flow nasal cannula (HFNC), guided by clinical severity. Objective transfer triggers included: (1) requirement for invasive mechanical ventilation; and (2) complications necessitating advanced or surgical interventions unavailable locally.

## Study outcomes

The primary outcome was the ultimate triage decision (transferred versus locally managed) within the confirmed RDS subgroup, evaluating the predictive utility of baseline LUS aeration scores. Secondary outcomes included the overall distribution of baseline LUS aeration scores across the entire study cohort.

### Statistical analysis

Data were analyzed using SPSS 26.0. Continuous variables were presented as medians with interquartile ranges (IQR) and compared using the Mann-Whitney U test, as the data did not follow a normal distribution. Categorical variables were presented as frequencies and percentages (*n*, %) and compared using Fisher’s exact test.

To assess the predictive performance for neonatal triage, we applied a predefined high-risk threshold (LUS aeration score ≥ 10) based on existing literature [13–15]. Using this specific cut-off, we calculated the sensitivity, specificity, and negative predictive value (NPV) alongside their exact 95% confidence intervals (CIs). A two-sided *P* value < 0.05 was considered statistically significant.

## Results

### Study population and baseline characteristics

A total of 34 neonates (gestational age range: 30 to 40 + 2 weeks) were enrolled in this pilot cohort and stratified into confirmed RDS (*n* = 17) and non-RDS (*n* = 17) groups based on final discharge diagnoses (Table 1). Neonates in the confirmed RDS group were significantly more premature and had lower birth weights compared to the non-RDS cohort (both *P* ≤ 0.001). The RDS group exhibited significantly lower 1-minute and 5-minute Apgar scores (*P* = 0.003 and *P* = 0.019, respectively). Baseline LUS aeration scores demonstrated a broad distribution (median: 8; IQR 5.2–10.8). Other baseline demographics, including sex distribution and the incidence of small for gestational age (SGA), were comparable between the two groups (all *P* > 0.05).

**Table 1:** Demographic and clinical characteristics of the study population

Variables	Total (*n* = 34)	RDS group (*n* = 17)	Non-RDS group (*n* = 17)	*P* value
Demographics & Birth History				
Male sex, n (%)	15 (44%)	9 (53%)	6 (35%)	0.491
Gestational age (wk), median (IQR)	34.9 (33.9–37.2)	34.0 (33.0–34.7)	37.3 (34.9–39.7)	< 0.001
Birth weight (g), median (IQR)	2196 (1963–2658)	1990 (1830–2170)	2580 (2260–3230)	0.001
SGA, n (%)	4 (12%)	2 (12%)	2 (12%)	1.000
Antenatal corticosteroids, n (%)	12 (35%)	8 (47%)	4 (24%)	0.282
Apgar score at 1 min, median (IQR)	9 (8–9)	8 (8–9)	9 (9–10)	0.003
Apgar score at 5 min, median (IQR)	9 (9–10)	9 (9–10)	10 (9–10)	0.019
Clinical Interventions				
Initial non-invasive respiratory support (nCPAP/HFNC), n (%)	34 (100%)	17 (100%)	17 (100%)	1.000
Imaging Findings				
LUS aeration score, median (IQR)	8 (5.2–10.8)	10 (9–12)	5 (4–8)	N/A
Positive chest X-ray, n (%) *	25 (74%)	12 (71%)	13 (77%)	1.000

Values are presented as median (IQR) or number (%)

*Abbreviations*: *RDS* Respiratory distress syndrome, *IQR* Interquartile range, *SGA* Small for gestational age,* nCPAP* Nasal continuous positive airway pressure, *HFNC* High-flow nasal cannula; *LUS*, lung ultrasound,* N/A* not applicable

* A “positive chest X-ray” was defined as the presence of any abnormal radiographic finding. In the RDS group, this primarily included typical ground-glass opacities or air bronchograms; in the non-RDS group, it included findings consistent with transient tachypnea of the newborn, congenital pneumonia, or pneumothorax

*P* values were calculated using the Mann-Whitney U test for continuous variables and Fisher's exact test for categorical variables

### Triage Decisions in the Confirmed RDS Subgroup

Within the confirmed RDS subgroup, 6 neonates (35%) required tertiary transfer—indicated by the need for invasive mechanical ventilation (*n* = 4), severe pneumothorax (*n* = 1), and hemodynamically significant patent ductus arteriosus (hsPDA) (*n* = 1)—while 11 (65%) were successfully managed locally (Table 2). Overall, exogenous surfactant was administered to 13 neonates (76%).

**Table 2:** Comparison of clinical characteristics and LUS aeration scores between transferred and locally managed neonates within the RDS subgroup (*n* = 17)

Variables	Transferred (*n* = 6)	Locally managed (*n* = 11)	*P* value
Demographics			
Gestational age (wk), median (IQR)	32.6 (31.5–33.8)	34.1 (33.5–34.8)	0.098
Birth weight (g), median (IQR)	1860 (1520–2080)	2000 (1905–2325)	0.180
Clinical Interventions			
Exogenous pulmonary surfactant, *n* (%)	5 (83%)	8 (73%)	1.000
Imaging Findings			
LUS aeration score, median (IQR)	12 (10–14)	10 (8.5–11)	0.242
CXR Positive (n%) *	6 (100%)	6 (55%)	0.102

Values are presented as median (IQR) or number (%)

*Abbreviations*: *RDS* Respiratory distress syndrome, *IQR* Interquartile range, *LUS *Lung ultrasound, *CXR* chest X-ray

* A “positive chest X-ray” was defined as the presence of any abnormal radiographic finding. In this RDS subgroup, this primarily included typical ground-glass opacities, air bronchograms, or white lung

*P* values were calculated using the Mann-Whitney U test for continuous variables and Fisher’s exact test for categorical variables

**Fig. 1:**
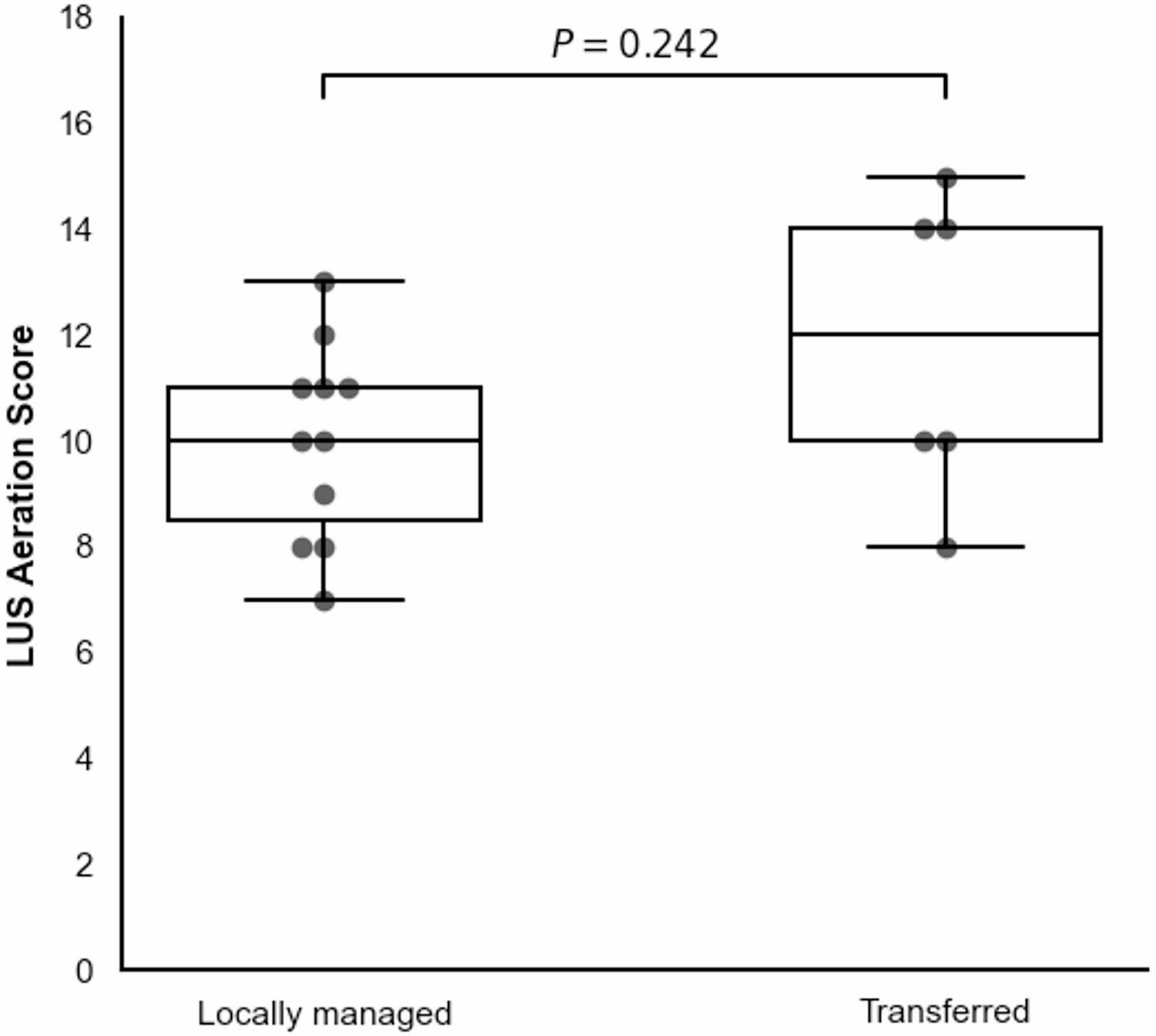
Comparison of baseline LUS aeration scores between locally managed and transferred neonates. The boxplots illustrate the median and interquartile ranges (IQR), with whiskers extending to the minimum and maximum values. Individual data points are superimposed as a swarm plot to display the full distribution (*n* = 17). Statistical comparison was performed using the Mann-Whitney U test, revealing no significant difference between the two clinical trajectories (*P* = 0.242)

Although transferred neonates exhibited a higher median baseline LUS aeration score (12 vs. 10), this trend was not statistically significant (*P* = 0.242). The full distribution of baseline scores stratified by triage outcome is illustrated in Figure 1. Applying the LUS aeration score ≥ 10 yielded a high sensitivity of 83.3% (95% CI: 43.6%–97.0%) and a specificity of 36.4% (95% CI: 15.2%–64.6%) for predicting transfer. The negative predictive value (NPV) for ruling out transfer in neonates with scores < 10 was 80.0% (95% CI: 37.6%–96.4%). The single false-negative case required transfer due to the development of severe pneumothorax. 

## Discussion

This pilot study demonstrates the feasibility and clinical utility of bedside LUS for evaluating neonatal respiratory distress in a resource-limited county hospital. While baseline LUS aeration scores showed an elevated trend in the transfer group (12 vs. 10), the statistical difference was not significant (*P* = 0.242). However, a critical novel finding emerges when evaluating LUS aeration score as a triage tool using a predefined high-risk threshold (≥ 10). While previous studies have thoroughly validated this cut-off for predicting surfactant therapy [13–15], our study innovatively translates this physiological metric into a logistical triage endpoint—the need for pre-emptive tertiary transfer due to anticipated advanced respiratory and hemodynamic support.

Applying this threshold revealed an asymmetric triage utility, excelling as a “rule-out” tool. It yielded a high sensitivity (83.3%) and a notable negative predictive value (NPV) of 80.0%, safely empowering local physicians to retain neonates with scores < 10. Conversely, the relatively low specificity (36.4%)—which translates to a substantial proportion of “false-positive” cases (i.e., neonates with scores ≥10 managed locally)—aligns with real-world clinical dynamics. A score ≥10 does not dictate an absolute transfer mandate; rather, it acts as an early warning signal triggering immediate local interventions, such as exogenous surfactant. Because these timely treatments effectively alter the disease trajectory, ultimate transfer decisions are often circumvented and instead driven by subsequent cardiopulmonary complications (e.g., severe pneumothorax, hsPDA). Furthermore, the single false-negative case highlights an inherent limitation of aeration-based scoring: pneumothorax, which manifests as paradoxical A-line restoration in affected zones, may not elevate baseline scores despite representing a transfer-requiring emergency. 

By incorporating bedside LUS into a composite clinical-sonographic diagnostic workflow, county-level clinicians are empowered to effectively integrate imaging data with the neonate's overall physiological maturity. This positions the LUS aeration score not merely as a crude, automatic transfer trigger, but as a nuanced functional assessment tool. Ultimately, this rational triage approach provides local physicians with added confidence and supportive evidence to safely retain neonates, thereby optimizing regional medical resources and facilitates early mother-neonate bonding —a cornerstone of family-integrated care [16].

Beyond primary triage, the utility of the LUS aeration score extends seamlessly into acute bedside management. While tertiary neonatal centers actively integrate these scores to optimize surfactant timing and anticipate non-invasive support failure [2, 3], this capability is arguably more transformative in resource-limited settings. In county hospitals, delayed radiographic interpretations often hinder critical decision-making. The rapid, point-of-care nature of bedside LUS bypasses these traditional logistical bottlenecks, facilitating the confident execution of time-sensitive interventions directly at the incubator.

Regarding respiratory support, all baseline LUS assessments were performed exclusively during non-invasive support (nCPAP/HFNC), aligning with foundational validations of LUS aeration scores [10]. Because Hoshino et al. [17] noted that positive pressure from invasive mechanical ventilation mechanically alters intrinsic lung aeration, this approach avoided intubation confounders and accurately captured baseline pathophysiology. Consequently, future studies establishing LUS aeration scores thresholds must explicitly stratify by respiratory support type.

However, widespread LUS implementation in primary care faces distinct pragmatic challenges. First, acquiring sonographic proficiency requires structured training. In our cohort, board-certified sonographers achieved competency through rigorous, guideline-based internal training. While this demonstrates the feasibility of cross-training, the lack of formalized quality control in understaffed facilities may challenge inter-observer consistency [18]. Second, device availability remains a hurdle [19]. Lacking a dedicated bedside point-of-care (POCUS) device, we relied on a shared, cart-based system transported from the central ultrasound department. Nonetheless, this professional-grade system featured high-resolution linear transducers essential for accurate scoring, underscoring that high-quality neonatal LUS is achievable through inter-departmental collaboration.

Several limitations exist. First, the small sample (*n* = 34) and underpowered RDS subgroup (*n* = 17) preclude multivariable regression. Importantly, while our evaluation of the ≥ 10 cut-off yielded a promising negative predictive value (NPV) and high sensitivity, the small sample size resulted in notably wide 95% confidence intervals. These metrics should be regarded as preliminary effect size estimates rather than definitive triage criteria. Nevertheless, they provide a necessary methodological foundation for future, adequately powered multicenter trials. Furthermore, this restricted cohort size increases the risk of a Type II error regarding the non-significant differences in baseline scores (*P* = 0.242). Second, resource constraints precluded routine cord blood gas analysis and longitudinal tracking of outcomes like BPD; however, this does not diminish our acute triage findings. Finally, real-time scoring by a single physician precluded formal inter-observer variability calculations, highlighting the need for independent offline interpretations in future trials.

In conclusion, bedside LUS is a feasible tool for assessing the severity of neonatal respiratory distress in resource-limited settings. Specifically, the promising negative predictive value of the lung aeration score provides an objective basis and clinical confidence for managing low-risk neonates locally, thereby optimizing the allocation of healthcare resources. Further large-scale studies are needed to validate these preliminary findings.

## Supplementary Information

Supplementary Material 1.

## Data Availability

The datasets used and/or analysed during the current study are available from the corresponding author on reasonable request.

## Abbreviations

RDS: Respiratory distress syndrome
IQR: Interquartile range
SGA: Small for gestational age
nCPAP: Nasal continuous positive airway pressure
HFNC: high-flow nasal cannula
LUS: lung ultrasound
N/A: Not applicable

## References

[1] Blencowe H, Cousens S, Oestergaard MZ, Chou D, Moller AB, Narwal R, et al. National, regional, and worldwide estimates of preterm birth rates in the year 2010 with time trends since 1990 for selected countries: a systematic analysis and implications. Lancet. 2012;379:2162–72. 22682464 10.1016/S0140-6736(12)60820-4

[2] Luo K, Wang H, Huang F, Tang J. Optimal timing and cutoff range of lung ultrasound in predicting surfactant administration in neonates: A meta-analysis and systematic review. PLoS ONE. 2023;18(7):e0287758. 37498845 10.1371/journal.pone.0287758 PMC10374100

[3] Capasso L, Pacella D, Migliaro F, De Luca D, Raimondi F. Can lung ultrasound score accurately predict the need for surfactant replacement in preterm neonates? A systematic review and meta-analysis protocol. PLoS ONE. 2021;16(7):e0255332. 34320032 10.1371/journal.pone.0255332 PMC8318286

[4] Pezza L, Alonso-Ojembarrena A, Elsayed Y, Yousef N, Vedovelli L, Raimondi F, et al. Meta-Analysis of Lung Ultrasound Scores for Early Prediction of Bronchopulmonary Dysplasia. Ann Am Thorac Soc. 2022;19(4):659–67. 34788582 10.1513/AnnalsATS.202107-822OC

[5] Han C, Wang X, Pan M, Huang X. Diagnostic accuracy of lung ultrasound score for bronchopulmonary dysplasia in preterm neonates: a systematic review and meta-analysis. Front Pediatr. 2025;13:1694150. 41480379 10.3389/fped.2025.1694150 PMC12753949

[6] Li X, Wang H, Liu Y, Han T, Zhu X, Guo Y, et al. A nationwide survey on the management of neonatal respiratory distress syndrome: insights from the MUNICH survey in 394 Chinese hospitals. Ital J Pediatr. 2024;50:160. 39244592 10.1186/s13052-024-01741-7 PMC11380405

[7] Sharma D, Kumar P, Bansal A, Farahbakhsh N, Shastri S, Sharma P, et al. The role of chest X-ray in the diagnosis of neonatal respiratory distress syndrome: a systematic review concerning low-resource birth scenarios. Glob Health Action. 2024;17:2338633. 38660779 10.1080/16549716.2024.2338633 PMC11047214

[8] Fenton TR, Elmrayed S, Alshaikh BN. Fenton Third-Generation Growth Charts of Preterm Infants Without Abnormal Fetal Growth: A Systematic Review and Meta-Analysis. Paediatr Perinat Epidemiol. 2025;39(6):543–55. 40534585 10.1111/ppe.70035 PMC12391854

[9] Sweet DG, Carnielli VP, Greisen G, Hallman M, Klebermass-Schrehof K, Ozek E, et al. European consensus guidelines on the management of respiratory distress syndrome: 2022 update. Neonatology. 2023;120:3–23. 36863329 10.1159/000528914 PMC10064400

[10] Brat R, Yousef N, Klifa R, Reynaud S, Shankar Aguilera S, De Luca D. Lung Ultrasonography score to evaluate oxygenation and surfactant need in neonates treated with continuous positive airway pressure. JAMA Pediatr. 2015;169:e151797. 26237465 10.1001/jamapediatrics.2015.1797

[11] Raimondi F, Yousef N, Rodriguez-Fanjul J, De Luca D, Corsini I, Dani C, et al. Point-of-care lung ultrasound in neonatology: classification into descriptive and functional applications. Pediatr Res. 2018;84:631–7. 10.1038/s41390-018-0114-9 PMC7094915 30127522

[12] Mongodi S, Cortegiani A, Alonso-Ojembarrena A, Biasucci DG, Bos LDJ, Bouhemad B, et al. ESICM-ESPNIC international expert consensus on quantitative lung ultrasound in intensive care. Intensive Care Med. 2025;51(6):1022–49. 40353867 10.1007/s00134-025-07932-y

[13] Gupta D, Priyadarshi M, Chaurasia S, Singh P, Basu S. Lung ultrasound for prediction of surfactant requirement in Indian preterm neonates: a diagnostic accuracy study. Eur J Pediatr. 2024;183(8):3599–606. 38829378 10.1007/s00431-024-05626-z

[14] Raimondi F, Migliaro F, Corsini I, Meneghin F, Pierri L, Salomè S, et al. Neonatal Lung Ultrasound and Surfactant Administration: A Pragmatic, Multicenter Study. Chest. 2021;160(6):2178–86. 34293317 10.1016/j.chest.2021.06.076

[15] Chan B, Torsitano C, Gordon S, Konana O, Singh Y. Substantiating and Adopting Lung Ultrasound Scores to Predict Surfactant Need in Preterm Neonates with Respiratory Distress Syndrome within an Institution. Am J Perinatol. 2024;41(12):1652–9. 38346693 10.1055/s-0044-1779500

[16] Franck LS, Waddington C, O’Brien K. Family Integrated Care for Preterm Infants. Crit Care Nurs Clin North Am. 2020;32(2):149–65. 32402313 10.1016/j.cnc.2020.01.001

[17] Hoshino Y, Futatani T, Kitamura S, Sato T, Maruo K, Yukitake Y, Lung ultrasound for confirmed surfactant deficiency (LUCID) study group. Lung ultrasound for predicting surfactant administration in ventilated and non-ventilated infants with respiratory distress syndrome: a multicenter prospective study. Eur J Pediatr. 2025;184(12):742. 10.1007/s00431-025-06580-0. Erratum in: Eur J Pediatr. 2025;184(12):790. 41212310 10.1007/s00431-025-06580-0

[18] Corsini I, Ficial B, Ciarcià M, Capasso L, Migliaro F, Rodriguez-Fanjul J, et al. Lung ultrasound scores in neonatal clinical practice: A narrative review of the literature. Pediatr Pulmonol. 2022;57(5):1157–66. 35229487 10.1002/ppul.25875

[19] Mazmanyan P, Kerobyan V, Shankar-Aguilera S, Yousef N, De Luca D. Introduction of point-of-care neonatal lung ultrasound in a developing country. Eur J Pediatr. 2020;179(7):1131–7. 32060800 10.1007/s00431-020-03603-w

