# Supplementary material for: Lung ultrasound for neonatal respiratory distress in a resource-limited county hospital: a pilot study
Source: BMC Pediatr. 2026 Mar 26;26:406. doi: 10.1186/s12887-026-06784-9 (PMC13141376; doi:10.1186/s12887-026-06784-9)
Supplement: Supplementary file 1 — Supplementary Material 1. [file 12887_2026_6784_MOESM1_ESM.docx]

**Supplementary Figure.**


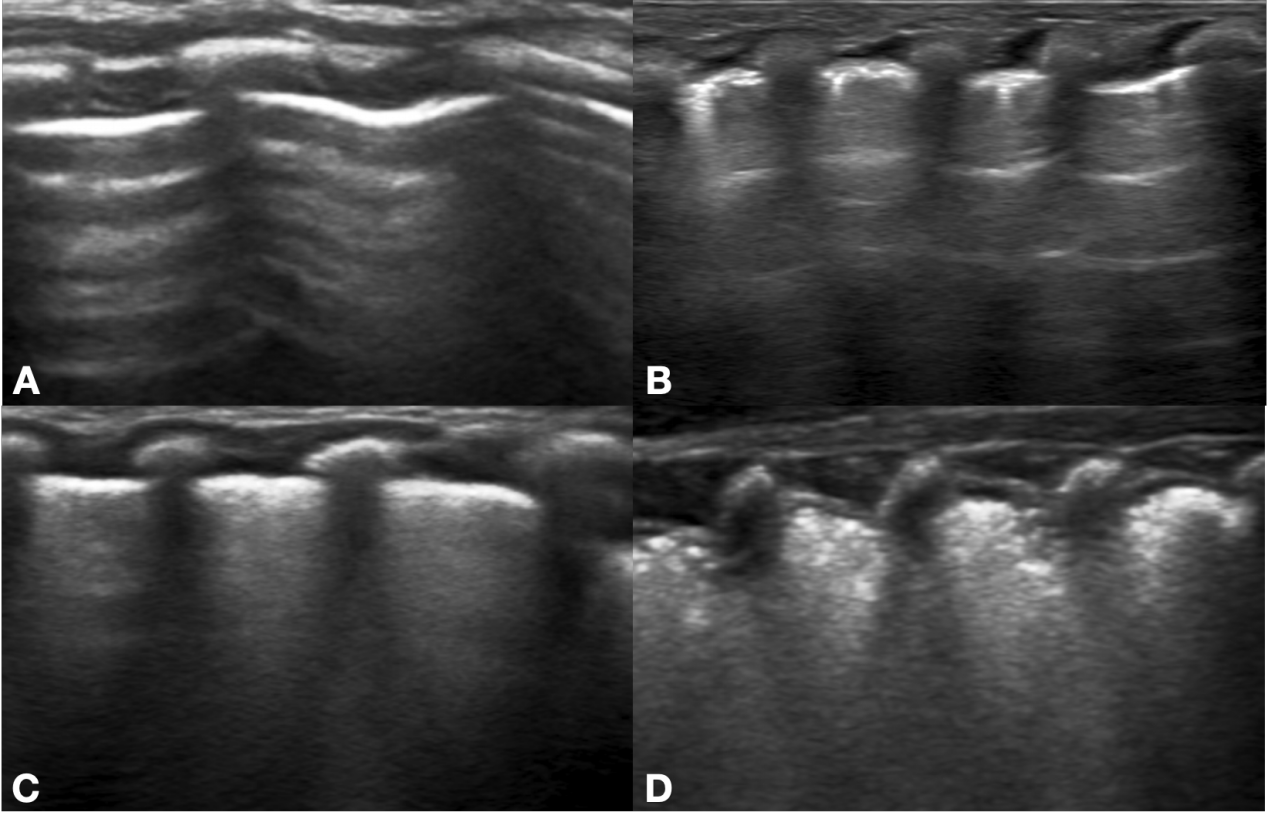


Representative lung ultrasound images illustrating the four-grade aeration scoring system. (A) Score 0: normal A-lines indicating normal aeration; (B) Score 1: three or more well-spaced B-lines indicating mild aeration loss; (C) Score 2: coalescent B-lines indicating moderate aeration loss; (D) Score 3: extended consolidation with air bronchograms indicating severe aeration loss. Each of the six lung zones was scored independently using this system, yielding a total LUS aeration score of 0–18.
